# Hypermethylated *MAL *gene – a silent marker of early colon tumorigenesis

**DOI:** 10.1186/1479-5876-6-13

**Published:** 2008-03-17

**Authors:** Guro E Lind, Terje Ahlquist, Matthias Kolberg, Marianne Berg, Mette Eknæs, Miguel A Alonso, Anne Kallioniemi, Gunn I Meling, Rolf I Skotheim, Torleiv O Rognum, Espen Thiis-Evensen, Ragnhild A Lothe

**Affiliations:** 1 Department of Cancer Prevention, Institute for Cancer Research, The Norwegian Radium Hospital, Rikshospitalet University Hospital, Oslo, Norway; 2 Centre for Cancer Biomedicine, University of Oslo, Oslo, Norway; 3 Centro de Biología Molecular Severo Ochoa, Consejo Superior de Investigaciones Científicas y Universidad Autónoma de Madrid, Madrid, Spain; 4 Laboratory of Cancer Genetics, Institute of Medical Technology, Tampere University Hospital and University of Tampere, Tampere, Finland; 5 Department of Urology, Akershus University Hospital, Lørenskog, Norway; 6 Faculty of Medicine, University of Oslo, Oslo, Norway; 7 Institute of Forensic Medicine, Rikshospitalet University Hospital, Oslo, Norway; 8 Medical Department, Rikshospitalet University Hospital, Oslo, Norway

## Abstract

**Background:**

Tumor-derived aberrantly methylated DNA might serve as diagnostic biomarkers for cancer, but so far, few such markers have been identified. The aim of the present study was to investigate the potential of the *MAL *(T-cell differentiation protein) gene as an early epigenetic diagnostic marker for colorectal tumors.

**Methods:**

Using methylation-specific polymerase chain reaction (MSP) the promoter methylation status of *MAL *was analyzed in 218 samples, including normal mucosa (n = 44), colorectal adenomas (n = 63), carcinomas (n = 65), and various cancer cell lines (n = 46). Direct bisulphite sequencing was performed to confirm the MSP results. *MAL *gene expression was investigated with real time quantitative analyses before and after epigenetic drug treatment. Immunohistochemical analysis of MAL was done using normal colon mucosa samples (n = 5) and a tissue microarray with 292 colorectal tumors.

**Results:**

Bisulphite sequencing revealed that the methylation was unequally distributed within the *MAL *promoter and by MSP analysis a region close to the transcription start point was shown to be hypermethylated in the majority of colorectal carcinomas (49/61, 80%) as well as in adenomas (45/63, 71%). In contrast, only a minority of the normal mucosa samples displayed hypermethylation (1/23, 4%). The hypermethylation of *MAL *was significantly associated with reduced or lost gene expression in *in vitro *models. Furthermore, removal of the methylation re-induced gene expression in colon cancer cell lines. Finally, MAL protein was expressed in epithelial cells of normal colon mucosa, but not in the malignant cells of the same type.

**Conclusion:**

Promoter hypermethylation of *MAL *was present in the vast majority of benign and malignant colorectal tumors, and only rarely in normal mucosa, which makes it suitable as a diagnostic marker for early colorectal tumorigenesis.

## Background

Epigenetic changes – non-sequence-based alterations that are inherited through cell division [1] – are frequently seen in human cancers, and likewise as genetic alterations they may lead to disruption of gene function. In colorectal cancer, several tumour suppressor genes have been identified to be epigenetically inactivated by CpG island promoter hypermethylation, including the DNA mismatch repair gene *MLH1 *[2-4], the gatekeeper *APC *[5], and the cell cycle inhibitor *CDKN2A *[6], to mention some. In addition to contributing to, or accompanying, the stepwise development of malignant colorectal carcinomas from benign adenomas, aberrant DNA methylation holds great promises for cancer diagnostics [7]. Based on the ubiquity of aberrant promoter methylation and the ability to detect this methylated DNA in body fluids, such as blood, the presence of this altered DNA may represent potential diagnostic biomarkers for cancer. For non-invasive detection of colorectal tumours, stool is the obvious source of DNA for such investigations and several studies have identified cancer-derived aberrant DNA hypermethylation using this approach [8-10]. However, the sensitivity and specificity of these tests are still suboptimal and would benefit from incorporating additional biomarkers.

We recently published a list of promising novel target genes for hypermethylation in colorectal tumours [11]. Among these was the *MAL *(T-cell differentiation protein) gene, and we then communicated that the CpG rich promoter of *MAL *seemed to be hypermethylated in the majority of colorectal tumours [12].

The *MAL *gene, which was initially isolated and cloned in 1987, maps to chromosome band 2cen-q13, encodes a 17 kDa integral membrane protein, and contains a CpG island [13,14]. Originally, expression of *MAL *was found in intermediate and late stages of T-cell differentiation, and MAL was suggested to play a role in membrane signalling [15]. In recent years, MAL has also been shown to play a role in apical transport, which is a polarized transport of lipids and proteins to the apical (external facing) membrane in certain cell types [16]. Such polarized transport is essential for the proper functioning of epithelial cells, and the neoplastic transformation process is frequently associated with loss of this polarized phenotype [16]. Finally, MAL has been shown to possess tumour suppressor capabilities by suppressing motility, invasion, and tumorigenicity and enhance apoptosis in oesophageal cancer [17].

In the present study, we have compared the promoter methylation status of *MAL *in a large series of normal colorectal mucosa samples, with those of benign and malignant colorectal tumours. Furthermore, RNA and/or protein expression levels of MAL were determined in *in vivo *tumours as well as in *in vitro *models, the latter also including various cancer types. The findings were used to decide whether or not methylated *MAL *is suitable as a diagnostic marker for early colorectal tumorigenesis.

## Methods

### Patients and cell lines

DNA from 218 fresh-frozen samples was subjected to methylation analysis, including 65 colorectal carcinomas (36 micro satellite stable; MSS, and 29 with micro satellite instability; MSI) from 64 patients, 63 adenomas, median size 8 mm, range 5–50 mm (61 MSS and 2 MSI) from 52 patients, 21 normal mucosa samples from 21 colorectal cancer patients (taken from distant sites from the primary carcinoma), and another 23 normal colorectal mucosa samples from 22 cancer-free individuals, along with 20 colon cancer cell lines (11 MSS and 9 MSI), and 26 cancer cell lines from various tissues (breast, kidney, ovary, pancreas, prostate, and uterus; Table 1). The mean age at diagnosis was 70 years (range 33 to 92) for patients with carcinoma, 67 years (range 62 to 72) for persons with adenomas, 64 years (ranging from 24 to 89) for the first group of normal mucosa donors, and 54 years (ranging from 33 to 86) for the second group of normal mucosa donors. The colorectal carcinomas and normal samples from cancer patients were obtained from an unselected prospective series collected from seven hospitals located in the South-East region of Norway [18]. The adenomas were obtained from individuals attending a population based sigmoidoscopic screening program for colorectal cancer [19]. The normal mucosa samples from cancer-free individuals were obtained from deceased persons, and the majority of the total set of normal samples (27/44) consisted of mucosa only, whereas the remaining samples were taken from the bowel wall. Additional clinico-pathological data for the current tumour series include gender and tumour location, as well as polyp size and total number of polyps per individual for the adenoma series.

**Table 1:** Promoter methylation status of *MAL *in cell lines of various tissues.

**Cell line**	**Tissue**	**Promoter methylation status**	**Methylation frequency**
BT-20	Breast	M	57%
BT-474	Breast	U/M	
Hs 578T	Breast	U	
SK-BR-3	Breast	U	
T-47D	Breast	U/M	
ZR-75-1	Breast	U	
ZR-75-38	Breast	M	

Co115	Colon	M	95%
HCT15	Colon	M	
HCT116	Colon	M	
LoVo	Colon	M	
LS174T	Colon	M	
RKO	Colon	M	
SW48	Colon	M	
TC7	Colon	M	
TC71	Colon	M	
ALA	Colon	M	
Colo320	Colon	M	
EB	Colon	M	
FRI	Colon	U/M	
HT29	Colon	M	
IS1	Colon	M	
IS2	Colon	M	
IS3	Colon	M	
LS1034	Colon	M	
SW480	Colon	M	
V9P	Colon	U	

ACHN	Kidney	U	50%
Caki-1	Kidney	U	
Caki-2	Kidney	M	
786-O	Kidney	U/M	

ES-2	Ovary	U/M	50%
OV-90	Ovary	U/M	
Ovcar-3	Ovary	U	
SK-OV-3	Ovary	U	

AsPC-1	Pancreas	M	67%
BxPC-3	Pancreas	U	
CFPAC-1	Pancreas	U	
HPAF-II	Pancreas	M	
PaCa-2	Pancreas	M	
Panc-1	Pancreas	U/M	

LNCaP	Prostate	U	0%

AN3 CA	Uterus	U/M	75%
HEC-1-A	Uterus	M	
KLE	Uterus	U	
RL95-2	Uterus	M	

The promoter methylation status of the individual cell lines was assessed by methylation-specific polymerase chain reaction (MSP). The methylation frequency reflects the number of methylated (M and U/M) samples from each tissue. Abbreviations: U, unmethylated; M, methylated.

All samples were retrieved from approved research biobanks and are part of research projects approved according to national guidelines (Biobank; registered at the Norwegian Institute of Public Health. Projects: Regional Ethics Committee and National Data Inspectorate).

Two colon cancer cell lines, HCT15 and HT29, were subjected to treatment with the demethylating drug 5-aza-2'deoxycytidine (1 μM for 72 h), the histone deactetylase inhibitor trichostatin A (0.5 μM for 12 h) and a combination of both (1 μM 5-aza-2'deoxycytidine for 72 h, 0.5 μM trichostatin A added the last 12 h).

### Bisulphite treatment and methylation-specific polymerase chain reaction (MSP)

DNA from primary tumours and normal mucosa samples was bisulphite treated as previously described [11,20], whereas DNA from colon cancer cell lines was bisulphite treated using the EpiTect bisulphite kit (Qiagen Inc., Valencia, CA, USA). The promoter methylation status of *MAL *was analyzed by methylation-specific polymerase chain reaction (MSP) [21], using the HotStarTaq DNA polymerase (Qiagen). All results were confirmed with a second independent round of MSP. Human placental DNA (Sigma Chemical Co, St. Louis, MO, USA) treated *in vitro *with *Sss1 *methyltransferase (New England Biolabs Inc., Beverly, MA, USA) was used as a positive control for the methylated MSP reaction, whereas DNA from normal lymphocytes was used as a positive control for unmethylated alleles. Water was used as a negative control in both reactions. The primers were designed with MethPrimer [22] and their sequences are listed in Table 2, along with the product fragment lengths and primer locations.

**Table 2:** PCR primers used for MSP and bisulphite sequencing.

**Primer set**	**Sense primer**	**Antisense primer**	**Frg. Size, bp**	**An. Temp**	Fragment location*
MAL MSP-M	TTCGGGTTTTTTTGTTTTTAATTC	GAAAACCATAACGACGTACTAACGT	139	56	-71 to 68
MAL MSP-U	TTTTGGGTTTTTTTGTTTTTAATTT	ACAAAAACCATAACAACATACTAACATC	142	56	-72 to 70
MAL BS_A	GGGTTTTTTTGTTTTTAATT	ACCAAAAACCACTCACAAACTC	236	53	-68 to 168
MAL BS_B	GGAAAAATGAAGGAGATTTAAATTT	AATAACCTAAACRCCCCC	404	50	-427 to -23

Abbreviations: MSP, methylation-specific polymerase chain reaction; BS, bisulphite sequencing; M, methylated-specific primers; U, unmethylated-specific primers; Frg. Size, fragment size; An. Temp, annealing temperature (in degrees celsius). *Fragment location lists the start and end point (in base pairs) of each fragment relative to the transcription start point provided by NCBI (RefSeq ID NM_002371).

### Bisulphite sequencing

All colon cancer cell lines (n = 20) were subjected to direct bisulphite sequencing of the *MAL *promoter [23]. Two fragments were amplified: fragment A, covering bases -68 to 168 relative to the transcription start point (overlapping with our MSP product), and fragment B covering bases -427 to -23. Fragment A covered altogether 24 CpG sites and was amplified using the HotStarTaq DNA polymerase and 35 PCR cycles. Fragment B covered altogether 32 CpG sites and was amplified using the same polymerase and 36 PCR cycles. The primer sequences are listed in Table 2. Excess primer and nucleotides were removed by ExoSAP-IT treatment following the protocol of the manufacturer (GE Healthcare, USB Corporation, Ohio, USA). The purified products were subsequently sequenced using the dGTP BigDye Terminator Cycle Sequencing Ready Reaction kit (Applied Biosystems, Foster City, CA, USA) in an AB Prism 3730 sequencer (Applied Biosystems). The approximate amount of methyl cytosine of each CpG site was calculated by comparing the peak height of the cytosine signal with the sum of the cytosine and thymine peak height signals, as previously described [24]. CpG sites with ratios ranging from 0 – 0.20 were classified as unmethylated, CpG sites within the range 0.21 – 0.80 were classified as partially methylated, and CpG sites ranging from 0.81 – 1.0 were classified as hypermethylated.

### cDNA preparation and real-time quantitative gene expression

Total RNA was extracted from cell lines (n = 46), tumours (n = 16), and normal tissue (n = 3) using Trizol (Invitrogen, Carlsbad, CA, USA) and the RNA concentration was determined using ND-1000 Nanodrop (NanoDrop Technologies, Wilmington, DE, USA). For each sample, total RNA was converted to cDNA using a High-Capacity cDNA Archive kit (Applied Biosystems), including random primers. *MAL *(Hs00242749_m1 and Hs00360838_m1) and the endogenous controls *ACTB *(Hs99999903_m1) and *GUSB *(Hs99999908_m1) were amplified separately in 96 well fast plates following the recommended protocol (Applied Biosystems), and the real time quantitative gene expression was measured by the 7900 HT Sequence Detection System (Applied Biosystems). All samples were analyzed in triplicate, and the median value was used for data analysis. The human universal reference RNA (containing a mixture of RNA from ten different cell lines; Stratagene) was used to generate a standard curve, and the resulting quantitative expression levels of *MAL *were normalized against the mean value of the two endogenous controls.

### Tissue microarray

For *in situ *detection of protein expression in colorectal cancers, a tissue microarray (TMA) was constructed, based on the technology previously described [25]. Embedded in the TMA are 292 cylindrical tissue cores (0.6 mm in diameter) from ethanol-fixed and paraffin embedded tumour samples derived from 281 individuals. Samples from the same patient series has been examined for various biological variables and clinical end-points [18,26-28]. In addition, the array contains normal tissues from kidney, liver, spleen, and heart as controls. Ethanol-fixed normal colon tissues from four persons with no known history of colorectal cancer were obtained separately.

### Immunohistochemical *in situ *protein expression analysis

Five μm thick sections of the TMA blocks were transferred onto glass slides for immunohistochemical analyses. The sections were deparaffinized in a xylene bath for 10 minutes and rehydrated via a series of graded ethanol baths. Heat-induced epitope retrieval was performed by heating in a microwave oven at full effect (850 W) for 5 minutes followed by 15 minutes at 100 W immersed in 10 mM citrate buffer at pH 6.0 containing 0.05% Tween-20. After cooling to room temperature, the immunohistochemical staining was performed according to the protocol of the DAKO Envision+™ K5007 kit (Dako, Glostrup, Denmark). The primary antibody, mouse clone 6D9 anti-MAL [29], was used at a dilution of 1:5000, which allowed for staining of kidney tubuli as positive control, while the heart muscle tissue remained unstained as negative control [30]. The slides were counterstained with haematoxylin for 2 minutes and then dehydrated in increasing grades of ethanol and finally in xylene. Results from the immunohistochemistry were obtained by independent scoring by one of the authors and a reference pathologist.

### Statistics

All *P *values were derived from two tailed statistical tests using the SPSS 13.0 software (SPSS, Chicago, IL, USA). Fisher's exact test was used to analyze 2 × 2 contingency tables. A 2 × 3 table and Chi-square test was used to analyze the potential association between quantitative gene expression of *MAL *and promoter methylation status. Samples were divided into two categories according to their gene expression levels: low expression included samples with gene expression equal to, or lower than, the median value across all cell lines or all tumours, high expression included samples with gene expression higher that the median. The methylation status was divided into three categories: unmethylated, partial methylation, and hypermethylated.

## Results

### Promoter methylation status of *MAL *in tissues and cell lines

**Figure 1:**
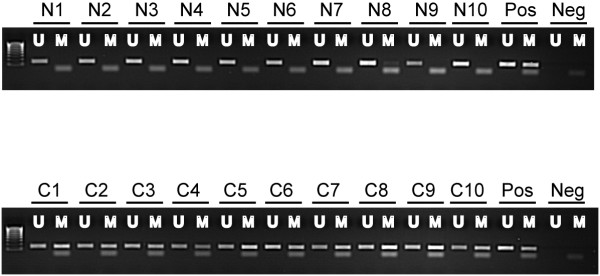
**Methylation status of the *MAL *promoter in normal colon mucosa samples and colorectal carcinomas**. Representative results from methylation-specific polymerase chain reaction are shown. A visible PCR product in lanes U indicates the presence of unmethylated alleles whereas a PCR product in lanes M indicates the presence of methylated alleles. N, normal mucosa; C, carcinoma; Pos, positive control (unmethylated reaction: DNA from normal blood, methylated reaction: *in vitro *methylated DNA); Neg, negative control (containing water as template); U, lane for unmethylated MSP product; M, lane for methylated MSP product.

The promoter methylation status of *MAL *was analyzed with MSP (Figure 1). One of 23 (4%) normal mucosa samples from non-cancerous donors and two of 21 (10%) normal mucosa samples taken in distance from the primary tumour were methylated but displayed only low-intensity band compared with the positive control after gel electrophoresis. Forty-five of 63 (71%) adenomas and 49/61 (80%) carcinomas showed promoter hypermethylation. Nineteen of twenty colon cancer cell lines (95%), and 15/26 (58%) cancer cell lines from various tissues (breast, kidney, ovary, pancreas, prostate, and uterus) were hypermethylated (Table 1 lists tissue-specific frequencies).

The hypermethylation frequency found in normal samples was significantly lower than in adenomas (*P *< 0.0001) and carcinomas (*P *< 0.0001). Hypermethylation of the *MAL *promoter was not associated with MSI status, gender, or age in neither malignant nor benign tumours. Among carcinomas, tumours with distal location in the bowel (left side and rectum) were more frequently hypermethylated than were tumours with proximal location, although not statistically significant (*P *= 0.088). Among adenomas, no significant association could be found between promoter methylation status of *MAL *and polyp size or number.

### Bisulphite sequencing verification of the promoter methylation status of *MAL*

**Figure 2:**
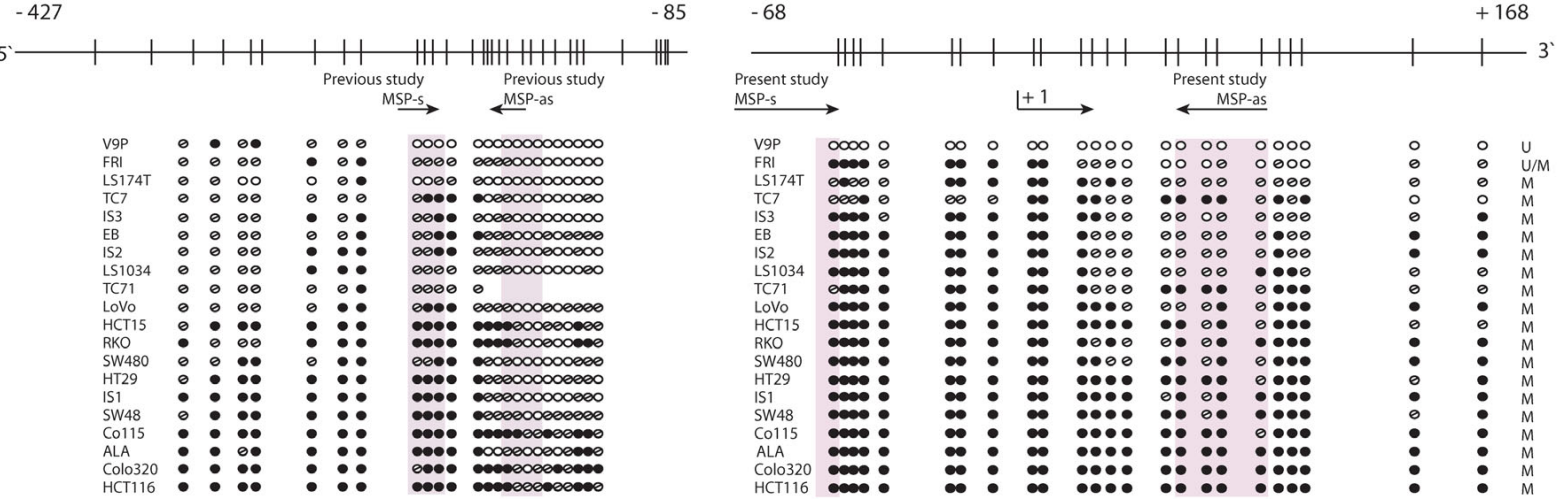
**Site specific methylation within the *MAL *promoter**. Bisulphite sequencing of the *MAL *promoter verifies methylation status assessed by methylation-specific polymerase chain reaction. The upper part of the figure is a schematic presentation of the CpG sites successfully amplified by the two analyzed bisulphite sequencing fragments, A (-68 to +168; to the right) and B (-427 to -85; to the left). The transcription start site is represented by +1 and the vertical bars indicate the location of individual CpG sites. The two arrows indicate the location of the MSP primers in the present study and a previously published study analyzing promoter methylation of *MAL *[31]. For the lower part of the figure, filled circles represent methylated CpGs; open circles represent unmethylated CpGs; and open circles with a slash represent partially methylated sites (the presence of approximately 20–80% cytosine, in addition to thymine). The column of U, M and U/M at the right side of this lower part lists the methylation status of the respective cell lines as assessed by us using MSP analyses. Abbreviations: MSP, methylation-specific PCR; s, sense; as, antisense; U, unmethylated; M, methylated; U/M, presence of both unmethylated and methylated band.

**Figure 3:**
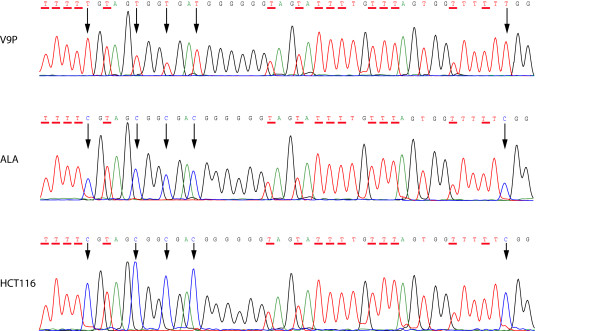
**The "bisulphite sequence" of the *MAL *promoter**. Representative bisulphite sequencing electropherograms of the *MAL *promoter in colon cancer cell lines. A subsection of the bisulphite sequence electropherogram, covering CpG sites +11 to +15 relative to transcription start. Cytosines in CpG sites are indicated by a black arrow, whereas cytosines that have been converted to thymines are underlined in red. The *MAL *promoter sequencing electropherograms illustrated here, are from the unmethylated V9P cell line and the hypermethylated ALA and HCT116.

Two overlapping fragments of the *MAL *promoter were bisulphite sequenced in 20 colon cancer cell lines. The results are summarized in Figure 2, and representative raw data can be seen in Figure 3. A good association was seen between the methylation status, as assessed by MSP, and the bisulphite sequences of the overlapping fragment A. However, in fragment B there was poor association with the MSP data. For this fragment, which is located farther upstream relative to the transcription start point, several consecutive CpG sites were frequently unmethylated and/or partially methylated. This held true also in cell lines shown to be heavily methylated around the transcription start point (fragment A; Figure 2).

### Real-time quantitative gene expression

**Figure 4:**
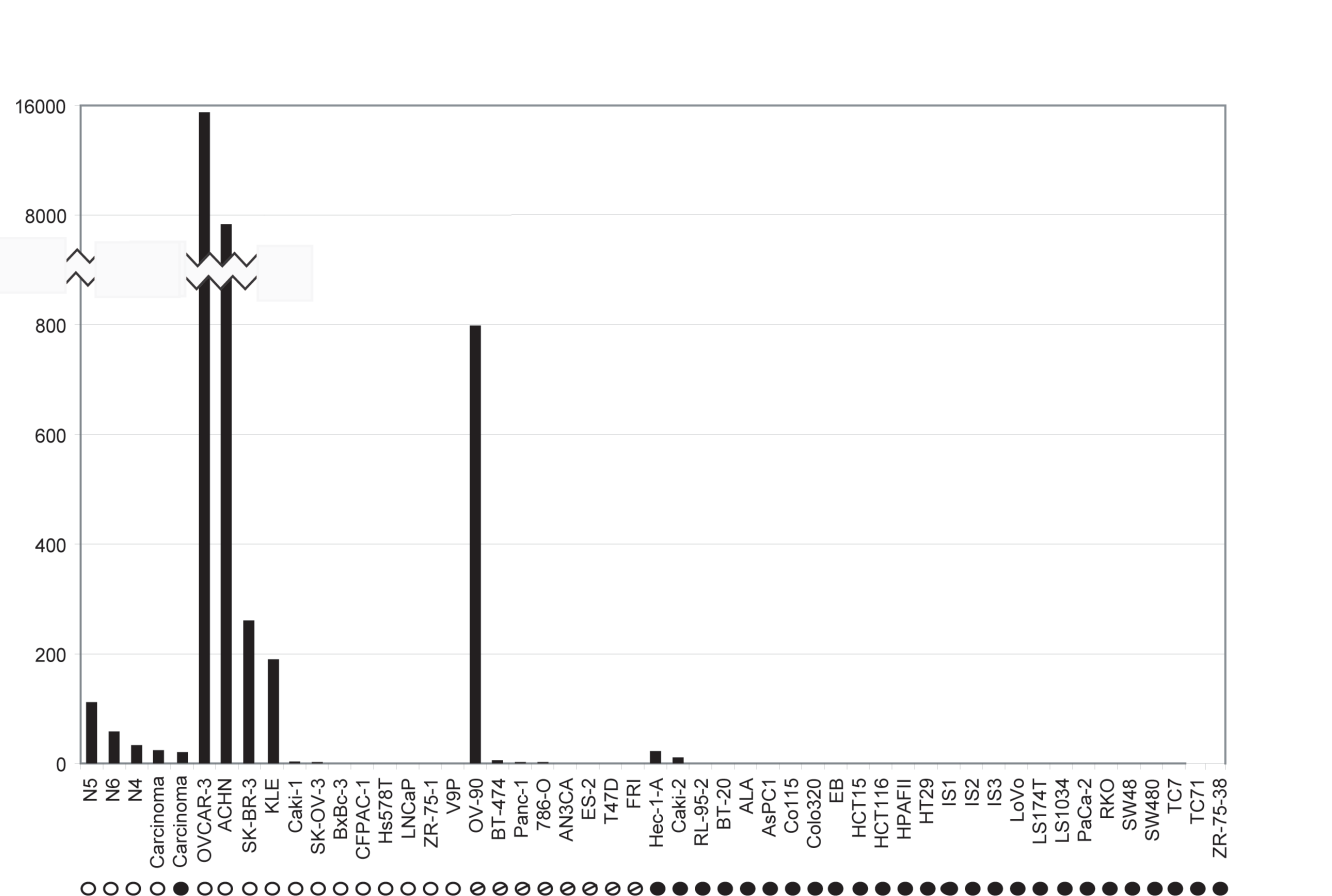
***MAL *expression in cancer cell lines and colorectal carcinomas**. Promoter hypermethylation of *MAL *was associated with reduced or lost gene expression in *in vitro *models. The quantitative gene expression level of *MAL *is displayed as a ratio between the average of two *MAL *assays (detecting various splice variants) and the average of the two endogenous controls, *GUSB *and *ACTB*. The value has been multiplied by a factor of 1000. Below each sample the respective methylation status is shown, as assessed by methylation-specific polymerase chain reaction. Filled circles represent promoter hypermethylation of *MAL*, open circles represent unmethylated *MAL*, and open circles with a slash represent the presence of both unmethylated and methylated alleles. Colorectal carcinomas are divided in an unmethylated group (n = 3) and a hypermethylated group (n = 13), and the median expression is displayed here. The tissue of origin for the individual cell lines can be found in table 1.

**Figure 5:**
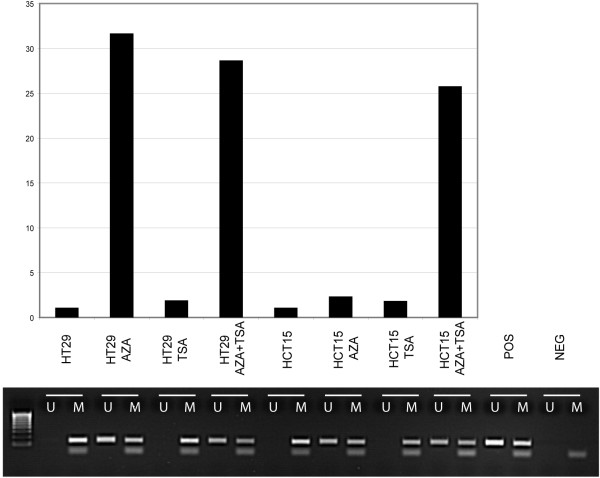
**Up-regulation of *MAL *expression after drug treatment**. Decreased promoter methylation of *MAL *followed by up-regulated mRNA expression in colon cancer cell lines was found after treatment with the demethylating 5-aza-2'deoxycytidine, alone and in combination with the deacetylase inhibitor trichostatin A. Upper panel demonstrate the relative expression values of *MAL *(linear scale) in two colon cancer cell lines, HT29 and HCT15, treated with 5-aza-2'deoxycytidine alone, trichostatin A alone, and the two drugs in combination. Lower panel illustrate *MAL *MSP results for the same samples. A visible PCR product in lanes U indicates the presence of unmethylated alleles whereas a PCR product in lanes M indicates the presence of methylated alleles. Abbreviation: AZA, 5-aza-2'deoxycytidine; TSA, trichostatin A; Pos, positive control (unmethylated reaction: DNA from normal blood, methylated reaction: *in vitro *methylated DNA); Neg, negative control (containing water as template); U, lane for unmethylated MSP product; M, lane for methylated MSP product.

The level of *MAL *mRNA expression in cell lines (n = 46), primary colorectal carcinomas (n = 16), and normal mucosa (n = 3) was assessed by quantitative real time PCR. There was a strong association between *MAL *promoter hypermethylation and reduced or lost gene expression among cell lines (*P *= 0.041; Figure 4). Furthermore, the gene expression of *MAL *was up-regulated in colon cancer cell lines after promoter demethylation induced by the combined treatment 5-aza-2'-deoxycytidine and trichostatin A (Figure 5). Treatment with the deacetylase inhibitor trichostatin A alone did not increase *MAL *expression, whereas treatment with the DNA demethylating 5-aza-2'-deoxycytidine led to high expression in HT29 cells, but more moderate levels in HCT15 cells (Figure 5). Among primary colorectal carcinomas, those harbouring promoter hypermethylation of *MAL *(n = 13) expressed somewhat lower levels of *MAL *mRNA compared with the unmethylated tumours (n = 3), although not statistically significant (Figure 4).

### MAL protein expression is lost in colorectal carcinomas

**Figure 6:**
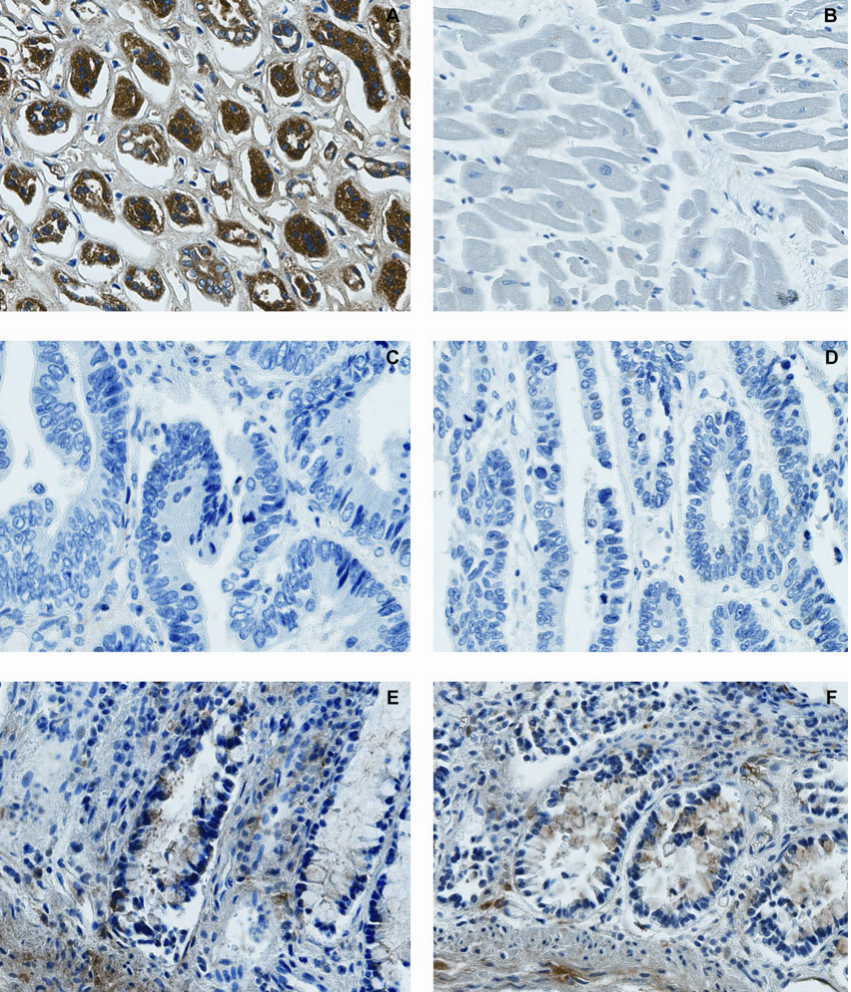
**Lack of MAL protein expression in colorectal carcinomas**. Positive cytoplasmic staining of MAL was found in kidney tubuli (A), and no staining was observed in heart muscle (B), in agreement with earlier reports [30]. The epithelial cells of colorectal carcinomas were MAL negative (C, D), whereas in normal colon tissue, cytoplasmic expression of MAL was found in both epithelia and connective tissue (E, F). All images were captured using the 40× lens (400× magnification).

To evaluate the immunohistochemistry analyses of MAL, kidney and heart muscle tissues were included as positive and negative controls, respectively (Figure 6A–B) [30]. From the 231 scorable colorectal tissue cores, *i.e. *those containing malignant colorectal epithelial tissue, 198 were negative for MAL staining (Figure 6C–D). Twenty-nine of these had positive staining in non-epithelial tissue components within the same tissue cores, mainly in neurons and blood vessels (not shown). In comparison, all the sections of normal colon tissue contained positive staining for MAL in the epithelial cells (Figure 6E–F).

## Discussion

In the present study, we have demonstrated that a sequence within the *MAL *promoter close to the transcription start is hypermethylated in the vast majority of malignant, as well as in benign colorectal tumours, in contrast to normal colon mucosa samples which are unmethylated, and we contend that *MAL *remains a promising diagnostic biomarker for early colorectal tumorigenesis [12]. The adenomas and carcinomas analyzed in the present study are from unselected clinical series and are therefore representative for the average risk population. However, the equal distribution between MSI and MSS carcinomas in the present study is not representative for a consecutive series.

Hypermethylation of *MAL *has, by quantitative methylation-specific polymerase chain reaction (MSP), previously been shown by others to be present only in a small fraction (6%, 2/34) of colon carcinomas [31], even though the expression of *MAL *was reported to be reduced/lost in the majority of colorectal tumours [11,17,31]. In contrast, we report here a significantly higher methylation frequency of *MAL *in both benign and malignant colorectal tumours (71% in adenomas and 80% in carcinomas). The discrepancy in methylation frequencies between the present report and the previous study by Mori and co-workers [31] is probably a consequence of study design. From direct bisulphite sequencing of colon cancer cell lines, we have now shown that the DNA methylation of *MAL *is unequally distributed within the CpG island of its promoter (Figure 2). CpG islands often span more than one kilobase of the gene promoter, and the methylation status within this region is sometimes mistakenly assumed to be equally distributed. This is exemplified by the *MLH1 *gene in which hypermethylation of a limited number of CpG sites approximately 200 base pairs upstream of the transcription start point invariably correlates with the lack of gene expression, while other sites do not [32,33]. Since the results of an MSP analysis rely on the match or mismatch of the unmethylated and methylated primer sequences to bisulphite treated DNA, one should ensure that the primers anneal to relevant CpG sites in the gene promoter. In the present study, we designed the MSP primers close to the transcription start point of the gene (-72 to +70) and found, by bisulphite sequencing, concordance between the overall methylation status of *MAL *as assessed by MSP and the methylation status of the individual CpG sites covered by our MSP primer set (Figure 2). This part of the CpG island was hypermethylated in the majority of colon cancer cell lines (95%). We also found that these cell lines, as well as those of other tissues, showed loss of *MAL *RNA expression from quantitative real time analyses, and that removal of DNA hypermethylation by the combined treatment of 5-aza-2'-deoxycytidine and Trichostatin A re-induced the expression of *MAL *in colon cancer cell lines (Figure 5). Furthermore, by analyzing a large series of clinically representative samples by protein immunohistochemistry we confirmed that the expression of MAL was lost in malignant colorectal epithelial cells as compared to normal mucosa.

We have further analyzed the same region of the *MAL *promoter as Mori *et al.*, which is located -206 to -126 base pairs upstream of the transcription start point [31]. By direct bisulphite sequencing, we showed that only a minority of the CpG sites covered by the Mori antisense primer were methylated in the 19 colon cancer cell lines that were heavily methylated around the transcription start point (Figure 2). We therefore conclude that the very low (six percent) methylation frequency initially reported for *MAL *in colon carcinomas [31] is most likely a consequence of the primer design and choice of CpG sites to be examined.

Inactivating hypermethylation of the *MAL *promoter might be prevalent also in other cancer types where low expression of *MAL *has been shown not to correlate with allelic loss or somatic mutations in the *MAL *gene [34]. In the present study, hypermethylated *MAL *was found in cancer cell lines from breast, kidney, ovary, and uterus.

The present analyses of cancer cell lines from seven tissues indicate that the hypermethylation of a limited area in the proximity of the transcription start point of *MAL *is associated with reduced or lost gene expression. However, among colorectal carcinomas and cell lines, MAL protein and gene expression seemed to be lost or reduced in all samples, including the minority with unmethylated *MAL *promoters. This underlines that loss of the MAL protein might have an important function in colorectal tumorigenesis and we hypothesize that early during colorectal neoplasia the gene is turned off by epigenetic mechanisms other than DNA methylation. The DNA methylation is subsequently recruited to the MAL promoter to "seal" the unexpressed state. Hence, it needs to be established whether *MAL *promoter hypermethylation is a cause or a consequence of the observed loss of gene expression in colorectal tumors. This is interesting from a biological – but not necessarily a diagnostic – perspective. The distinction between the two is supported by the fact that one of the most promising diagnostic biomarkers for colorectal cancer reported so far, DNA hypermethylation of the vimentin (*VIM*) gene, is not expected to alter the gene expression, nor to confer a selective advantage upon cancer cells in the colon, considering the lack of *VIM *expression by normal colonic epithelial cells [9].

A sensitive non-invasive screening approach for colorectal cancer could markedly improve the clinical outcome for the patient. Such a diagnostic test could in principle measure the status of a single biomarker, although multiple markers are probably needed to achieve sufficient sensitivity and specificity. Several studies have successfully detected such tumour-specific products in the faeces, and most experience has been with mutant genetic markers, including *APC*, *KRAS*, *TP53*, and BAT-26 [35]. However, one of the most promising faecal DNA tests so far consisted of a combination of a genetic DNA integrity assay and an epigenetic *VIM *methylation assay, resulting in 88% sensitivity and 82% specificity [36]. This panel might be further improved by implementing *MAL *and/or, as suggested by others, the *SFRP2 *marker, which has an independent sensitivity and specificity of 77% in faecal DNA [8].

Hypermethylation of the *MAL *promoter represents, to the best of our knowledge, the most frequently hypermethylated gene among pre-malignant colorectal lesions, accompanied by low methylation frequencies in normal colon mucosa. The presence of such epigenetic changes in pre-malignant tissues might also have implications for cancer chemoprevention. By inhibiting or reversing these epigenetic alterations, the progression to a malignant phenotype might be prevented [37]. However, for the purpose of cancer risk assessment, *MAL *methylation status should be used in combination with other markers to recognize high risk adenomas.

## Conclusion

Promoter hypermethylation of *MAL *remains one of the most promising diagnostic biomarkers for early detection of colorectal tumours, and, together with other biomarkers, it merits further investigation with the purpose of developing a diagnostic marker panel with the necessary sensitivity and specificity to discover colorectal neoplasia and perform a risk assessment.

## Abbreviations

*MAL*, T-cell differentiation protein gene; MSI, micro satellite instability; MSP, methylation-specific polymerase chain reaction; MSS, micro satellite stable micro satellite instability; TMA, tissue microarray.

## Competing interests

The author(s) declare that they have no competing interests.

## Authors' contributions

GEL and TA carried out the MSP analyses and interpreted the results independent of each other. GEL additionally designed the study, carried out the bisulphite sequencing and the quantitative real-time PCR analyses, performed the statistics, and drafted the manuscript. MK carried out the immunohistochemistry analyses, interpreted the results together with an expert pathologist and contributed in manuscript preparations. MB isolated DNA from cancer cell lines. ME cultured all cell lines and treated colon cancer cell lines with epigenetic drugs. MAA provided the antibody for immunohistochemical analyses and contributed with scientific discussion. AK provided several of the cancer cell lines. GIM and TOR have collected the series of human primary carcinomas and normal mucosa tissue and provided all clinical and pathological information regarding these samples. RIS generated the tissue microarray and contributed in manuscript preparations. ETE has provided the series of adenoma samples and provided all clinical and pathological information regarding these samples and contributed in manuscript preparations. RAL conceived the study, participated in its design, contributed in evaluation of results, scientific discussion and in manuscript preparation. All authors have read and approved the final manuscript.
